# Electrophysiological activation by masked primes: Independence of prime-related and target-related activities

**DOI:** 10.2478/v10053-008-0008-1

**Published:** 2008-07-15

**Authors:** Werner Klotz, Manfred Heumann, Ulrich Ansorge, Odmar Neumann

**Affiliations:** 1 Department of Psychology, University of Bielefeld, Bielefeld, Germany; 2 Department of Cognitive Science and Department of Psychology, University of Osnabrueck, Osnabrueck, Germany

**Keywords:** metacontrast, EEG recording, LRP, Go/Nogo

## Abstract

Visual stimuli that are made invisible by metacontrast masking (primes) have a marked influence on behavioral and psychophysiological measures such as reaction time (RT) and the lateralized readiness potential (LRP). 4 experiments are reported that shed light on the effects that masked primes have on the LRP. Participants had a go-nogo task in which the prime was associated with 1 of 2 responses even if the target required participants to refrain from responding. To analyze the electrophysiological responses, we computed the LRP and applied an averaging method separating the activation due to the prime and the target. The results demonstrated that (a) masked primes activate responses even in a nogo situation, (b) this prime-related activation is independent of masking, (c) and is also independent of whether prime and target require the same responses (congruent condition) or different responses (incongruent condition).

## Introduction

Recent research has produced strong evidence that unconscious (“subliminal”) stimuli may affect behavior. Though there still are some doubts (e.g., Dulany, 2001) and discussions regarding methodological issues, such as the appropriate metric for the behavioral (indirect) measure (for recent discussions see Ansorge, Breitmeyer, & Becker, in press; Klauer & Greenwald, 2000; Miller, 2000; Schmidt & Vorberg, 2006), a consensus has emerged that nonconscious priming (or “perception without awareness”; Merikle, Smilek, & Eastwood, 2001) exists. One major open question is whether or not unconscious stimuli can have influences that require their semantic analysis (Damian, 2001; Kiefer, 2002; Kiefer & Spitzer, 2000; Kunde, Kiesel, & Hoffmann, 2003; Naccache & Dehaene, 2001; see also the study of Naccache et al., 2005, in which emotions were evoked by subliminal words). However, the large number of successful replications of the principal effect suggests that the effect is genuine.

**Figure 1.**
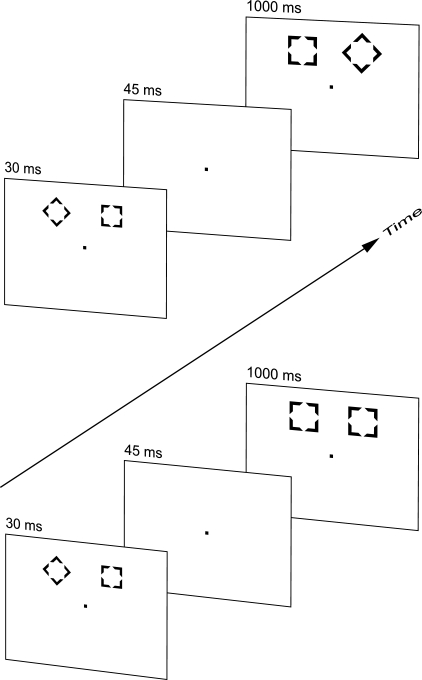
Sequence of stimulus events. The left half depicts an incongruent trial: The arrangement of the figures in the target pair is reversed with respect to the prime. The right half shows a masked nogo trial. In both cases, the diamond is assumed to be the target. Numbers designate stimulus duration.

One basic paradigm in the study of unconscious information processing is the metacontrast dissociation (see Figure 1; see also Klotz & Neumann, 1999; Klotz & Wolff, 1995; Neumann & Klotz, 1994). In this paradigm, participants have a two-choice reaction time (RT) task. In each trial they see a square and a diamond (a square rotated by 45°). One of these shapes is defined as the target, and participants are told to press the right button when the target appears on the right side of a monitor and the left button when it appears on the left side. The other shape serves as a distractor. Unknown to the participants, invisible primes are presented prior to the visible target-distractor pair. The shapes of the primes and the targets are designed so that the targets mask the primes by metacontrast masking (for examples and an explanation of metacontrast masking, see Breitmeyer, 1984; Breitmeyer & Ogmen, 2000,  2006).

The primes are also presented as a pair. To produce strong masking, they are similar in shape to the target stimuli, albeit smaller. Consider the case of a participant who is instructed to locate the diamond. If the square and the diamond in the prime pair are on the same sides as in the target pair, this is a congruent priming condition. If the arrangement is reversed, so that the diamond prime is at the position of the square mask, and the square prime is at the position of the diamond mask, we get an incongruent condition. Finally, in a neutral condition, neither of the prime stimuli is a target shape; both have the shape of a distractor. In the case of responding to diamonds, for example, this neutral prime pair consists of two squares.

Research with this paradigm (e.g., Ansorge, Klotz, & Neumann, 1998; Klotz & Wolff, 1995; Neumann & Klotz, 1994; Wolff, 1989 ) has consistently shown that, compared to RT in the neutral condition, RTs are shorter in the congruent condition and longer in incongruent trials. Participants also make more errors in the incongruent than in the neutral condition, whereas there are fewer errors in the congruent than in the neutral condition. If, however, subjects are required to discriminate the shapes of the primes, their performance does not deviate from chance, even after all sorts of precautions have been taken to improve their motivation, speed up, or slow down their judgments, etc. (Klotz & Neumann, 1999).

Besides the effects on RT and error rate, other studies with this method (e.g., Eimer, 1999; Jaśkowski, van der Lubbe, Schlotterbeck, & Verleger, 2002; Schlaghecken & Eimer, 2001) or similar paradigms (Leuthold & Kopp, 1998; Vath & Schmidt, 2007) have shown that the primes also exert a significant effect on the lateralized readiness potential (LRP). The LRP (for an overview, see Eimer, 1998) is a lateralized negativity that can be recorded from the scalp over the motor cortices prior to response execution. It is stronger over the motor cortex contralateral to the responding hand, and hence, the difference potential between the left and right hemispheres can be used as a measure of the selective preparation of a right or left hand response. The authors of the cited studies were able to show that the incongruent prime not only delayed the onset of the LRP, but that the incongruent LRP also deflected (“dipped”) in the direction of the primed response (e.g., see Figure 3, upper panel). This indicates that initially, a response is prepared that corresponds to the position of the target-similar shaped prime, even if the location of the target then ultimately demands the opposite response (see Jaśkowski et al., 2002, who discern motor from attentional lateralization).

These results demonstrate that masked stimuli impact on motor performance. They do not yet, however, indicate whether the primes have a target-independent influence. In principal, there are two possibilities: First, it could be that masking (that is the presentation of the target) affects only the conscious perception of the primes but leaves their effect on motor activation completely unaltered. In this case, one would expect identical effects of primes on the LRP, whether they are masked or not, and also independently of the motor-activation evoked by the targets. Alternatively, it could be that the motor response evoked by masked primes, while not being completely obliterated by the targets, is still modified by them.

In the former case, there would be a dissociation in the sense that masking affects conscious perception but not motor activation. In the latter case, there would be a dissociation in the sense that masking affects conscious perception more strongly than motor activation. Looking into these alternatives motivated the present study. More specifically, we ask whether or not there is an interaction between the prime-induced and the target-induced motor activation.

## Experiment 1

One limitation of the previous LRP studies on metacontrast dissociation has been that the LRP does not reveal how large the activation caused by the prime actually was. It must be assumed that the trace of the prime in the LRP (the “dip”, i.e., the target-ipsilateral activation in incongruent conditions) was in part influenced by activation caused by the target. However, the point in time at which target activation started and began to cancel out prime activation could not be assessed in any precise manner. By the same token, nothing could be said about the fate of prime-related activation after the LRP became dominated by target-related activity. There was a compound effect of both stimuli, with the effect of the prime merging into that of the target.

In the present experiments, we therefore intended to measure the prime’s influence on the LRP unconfounded by target-related motor activation. In addition to the congruent and incongruent conditions, there was a condition in which the visible stimuli provided no lateralized information. In this condition, after the prime pair, two visible distractors were shown instead of a target-distractor pair. Participants were instructed not to respond under these distractor conditions. Thus, any motor activation reflected in LRPs observed in this “nogo condition” must be caused by the prime. Furthermore, false alarms in these nogo trials, in particular, the responses to the side of the target-shaped prime, provided information about the ability of the prime to not only activate, but also trigger a response.

We applied an averaging method for the calculation of the LRP. The steps in obtaining the average prime and target activations entailed (a) averaging the data according to experimental condition and side of required response, which resulted in four averages (congruent/left, congruent/right, incongruent/left, and incongruent/right). Subsequently, these averages were (b) combined so that either prime-related activations or target-related activations cancel each other out. To obtain the measure of target activation, congruent/left target and incongruent/left target, and congruent/right target and incongruent/right target were combined, and the difference between these averages was computed. Because prime activation should reverse amplitude in incongruent/left target as compared to congruent/left target trials, the resulting LRP waveform will only contain target-related lateralization. The same holds true for incongruent/right target and congruent/right target conditions. Likewise, combining congruent/right target and incongruent/left target, on the one hand, and congruent/left target and incongruent/right target trials on the other hand and subtracting these waveforms from one another will reveal prime-related LRP activation, with target-related LRPs canceled out.

We thus obtained two independent measures of prime-related LRP activity: first, the directly measured prime-related activity under the nogo condition, and second, the calculated prime-related activity from the different prime-target combinations. If there are no differences between these two estimates of prime-related activity, then this would constitute strong evidence in favor of the hypothesis that prime-related activity is independent of target-related activity.

### Method

#### Participants, apparatus, and procedure

Nineteen participants (8 female, 11 male; mean age27 years) were recruited among the students at the University of Bielefeld. All had normal or corrected-to-normal vision. They participated in a single session of approximately 3 hr and were paid for their participation.

We used the stimuli of Klotz and Neumann (1999; see also Figure 1). In all the details ensuring that the participants could not have been aware of the primes (stimulus durations and intervals, shapes and sizes of stimuli, luminance, laboratory illumination, state of adaptation, instruction), the experiment was identical to those done by Klotz and Neumann, who showed that participants are not residually aware of the primes.

Stimuli were displayed black (0.5 cd/m^2^) on white (105 cd/m^2^) on a computer monitor with a refresh rate of 67 Hz, controlled by a PC that also recorded the behavioral data and triggered the EEG amplifier 100ms prior to the onset of the first stimulus. Stimuli appeared in pseudo-random order either above or below fixation with a retinal eccentricity of 3°. The stimuli that made up the mask pairs were 1.6° wide; the stimuli in the prime pairs were 1.1° wide. The two stimuli of both pairs were 2° apart, as measured from stimulus center to stimulus center. A fixation point was visible at the center of the screen during the entire experiment.

As shown in Figure 1, each trial started with the presentation of the prime pair, which was displayed for 30ms, either above or below fixation in a pseudo-random sequence. This pair was followed by a blank of 45ms. Hence, the stimulus onset asynchrony (SOA) was 75ms. Finally, the mask pair was displayed for 1,000 ms. The inter-trial interval varied randomly and was either 2,800 ms; 3,000 ms; or 3,200 ms. The prime pair always consisted of a diamond and a square, with the diamond on the left side in half of the trials. The mask pair consisted of a diamond and a square in 80% of the trials and of two distractors (either two squares or two diamonds, depending on which of the shapes was response-relevant) in the rest of the trials. Thus, one session of 800 trials comprised 160 nogo trials and 640 go trials, which in turn, contained 320 trials with congruent priming (target shape on the same side in prime pair and mask pair) and 320 incongruent trials (side of target shape reversed). After each block of 100 trials, the sequence was automatically interrupted for a break. Participants could also take a break during the inter-trial interval whenever they wished.

Participants sat in a dentist’s chair, with their horizontal line of gaze level with the center of the display. Digital joysticks, which could be moved left or right, were attached to both armrests. Nine participants were instructed to push the left joystick to the left if the diamond appeared on the left side of the mask pair, and to push the right joystick to the right if it appeared on the right. They were also instructed to refrain from any response when the diamond was not displayed and the mask pair consisted of only two squares. The other participants were assigned the square as their target. All were instructed to respond as quickly and as accurately as possible, and to refrain from eye movements and blinking while the stimuli were being displayed.

#### Electrophysiological recording

The EEG was recorded according to the international 10/20 system from electrodes Fz, Cz, Pz, Oz, C3’, and C4’ referenced to linked earlobes; the ground electrode was on the forehead. C3’ and C4’ designate sites 1 cm anterior to C3 and C4 locations. To control for artifacts, the electrooculogram (EOG) was recorded bipolarly from pairs of electrodes attached to the outer canthi of both eyes and to sites just above and below the right eye. The data were sampled at 250 Hz in epochs of 1 s with a low pass filter at 40 Hz (DC). Off-line, the data were baseline corrected relative to the first 100ms of each epoch. Artifact rejection included criteria of 50 µV for the EOG electrodes and 100 µV for the other electrodes. Trials in which at least one electrode showed no activity above 5 µV were also rejected.

#### Data reduction and analysis

 For the RT analysis, we computed the median of every subject and experimental go condition, and averaged those data. Trials with responses faster than 100 ms and slower than 1,000 ms were discarded. Responses in nogo trials were regarded as errors. Error rates were arc-sine-transformed before analysis (Winer, 1971). Error trials from all conditions were discarded from the EEG analysis. For the computation of the LRP waveforms, we used the averaging formula suggested by Coles (1989). We averaged both across the three conditions and across stimuli (primes vs. targets) in the go trials, yielding five waveforms. The nogo trials were averaged according to the side of the target-shaped prime. To obtain measures for the onsets of the waveforms, we repeatedly computed matched t-tests against 0 µV (a = .05), and used the first of four data points in a row that deviated from 0 µV. In the data reported, each LRP was subjected to this test. 

### Results

#### Reaction times and error rates

In the analysis of behavioral data, only those subjects were included who delivered useful EEG-data. Data were considered useful (a) if sufficient artifact-free sweeps were gained in each condition (> 100), and (b) if an LRP existed in the go conditions (squared systematical deviation in the LRP-area > squared random deviation in the pre-stimulus interval). In Experiment 1, none of the participants were excluded. Out of all trials, 0.3% were discarded because RTs were either faster than 100 ms or slower than 1,000 ms. Mean RTs were 409 ms in congruent trials and 439 ms in incongruent trials. Error rates were 0.9% and 2.8%, respectively. Participants responded in 4.7% of the nogo trials. Of these false alarms, the majority (3.7%) was to the side of the target-shaped prime. Matched *t*-tests revealed a significant priming effect for RTs, *t*(18) = 7.75, *p* < .01, and for error rates, *t*(18) = 2.72, *p* < .05, and a significant difference between the frequencies of false alarms in nogo trials to the side of the target-shaped prime and to the other side, *t*(18) = 2.12, *p* < .05.

#### Event-related potentials

**Figure 2.**
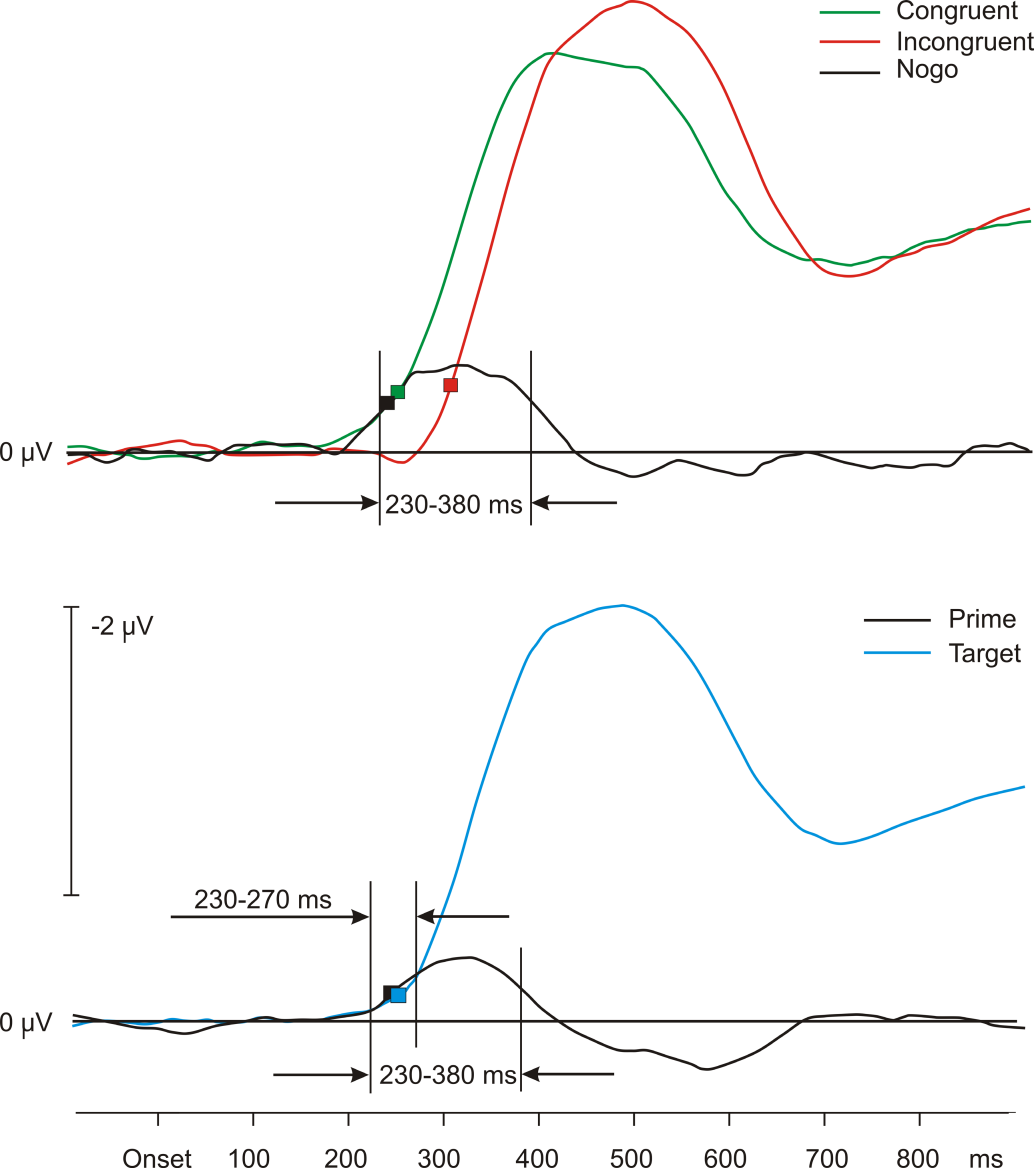
Upper panel: Grand average LRP waveforms of Experiment 1. Depicted are Nogo (masked), congruent, and incongruent conditions. Data were smoothed for this and all other figures, using floating means over 17 samples. The smoothing of the data concerns only the figures, not the statistical analyses. Lower panel: Grand average waveforms of the calculated LRP effects across conditions of prime and target in Experiment 1. Symbols stand for LRP onsets.

Out of all trials, 20% had to be excluded because of artifacts. Discarded trials were equally distributed across conditions. Figure 2 (upper panel) shows the grand average LRPs for the three conditions. The onsets of the waveforms obtained in congruent and incongruent trials qualitatively mimic the pattern of the RT data: The onset of the congruent waveform is at 232 ms, the onset of the incongruent waveform at 292 ms. The LRP of the nogo trials also deviated from baseline, starting at 224 ms.

The LRP data obtained by averaging across priming conditions (the calculated prime activity) are depicted in Figure 2 (lower panel). As expected, the influence of the target yielded the largest waveform, deviating from baseline at 240 ms. The prime’s influence was much smaller, its onset was at 232 ms. The mean amplitudes of the calculated prime and target activity were compared in a time window between 230270 ms. The activities in this interval do not differ statistically, -0.26 V and -0.18 V; *t*(18) = -1.26, *p* > .20. To find out whether the prime-related activities differed between go and nogo trials, we compared these calculated mean amplitudes in a time window between 230–380 ms with the measured prime activity in the nogo condition. The mean amplitude of the calculated prime activity of the go trials was -0.37 V, and the mean amplitude in nogo trials was -0.55 V. These values did not differ statistically, *t*(18) = 1.32, *p* > .20.

### Discussion

The behavioral data replicate standard findings, with increased RT latencies and error rates in the incongruent as compared to the congruent condition. The influence of the prime is also evident from the false alarm rates in the nogo condition, in which participants tended to produce the response to the side of the target-shaped prime.

The prime’s influence can also be clearly seen in the LRP of the nogo condition. The comparison of this waveform with the waveform that shows the calculated influence of the prime reveals a striking similarity. Though the nogo waveform necessarily contained more noise, since a maximum of only 160 trials per participant could be included, the two waveforms are remarkably similar. The LRP onsets are almost identical, and the mean LRP amplitudes over the whole course of the lateralization show no reliable difference.

Previous studies found an initial target-ipsilateral negativation in the incongruent condition (Eimer, 1999; Jaśkowski et al., 2002; Leuthold & Kopp, 1998). This “incongruity dip” is, however, absent in the present data. One possible explanation is that prime-related and target-related activities cancelled each other out in incongruent trials, thus eliminating the dip and delaying the onset of this waveform. This interpretation is illustrated in the analysis of the amplitudes of the increasing part of calculated prime- and target-related activities. These amplitudes did not differ, that is, a prime had the same impact as a target. This implies that, if the prime and the target began exerting their effects simultaneously, then the sum of target-evoked and prime-evoked lateralizations in the incongruent case should not diverge from baseline. The problem with this interpretation is, of course, that there was an SOA of 75 ms between primes and targets, which seems to imply that the prime’s effect started earlier than that of the target. (The reason might be that the processing of the target profits from the attention shift caused by the prime; see Neumann & Scharlau, 2007.)

One might conceive that the presence of a nogo condition has been a critical factor. Without such a condition, that is, in a standard two-choice RT task, response preparation can start as soon as information about the side of the required response is available, hence with prime presentation. By contrast, the go-nogo task might induce participants to postpone their response preparation until the target is available. To put it differently, the prime in a standard RT task can serve the purpose of both the stimulus that determines the side of the response and the purpose of an imperative stimulus. By contrast, only the target is apt to serve as the imperative stimulus in a task that includes a nogo condition.

This account, however, fails to explain that in the standard two-choice reaction task without nogo trials, target-evoked activity also overruled prime-evoked activity, as reflected in a large majority of the trials. Thus, factors besides the nogo condition are evidently responsible for shifting the weights of response activation toward the target and away from the prime. This argument notwithstanding, it is still possible that the nogo condition at least additionally delayed prime-induced response activation so that the otherwise notorious incongruity dip was eliminated. This was tested in Experiment 2, in which the nogo condition was omitted. We expected that the incongruity dip would be found under these task conditions.

Let us turn to the main purpose of Experiment 2. While there is evidence that the prime’s effect is independent of whether or not the target requires a response, the question remains whether the activation caused by the prime in congruent conditions is identical to the activation in incongruent conditions. Our estimate of activation by the prime in combination with go targets has been the average of congruent and incongruent conditions. It cannot, therefore, be decided from the present data whether the facilitation by a congruent prime is different from the inhibition by an incongruent prime.

This question is of some interest because response latencies and errors in previous studies (Klotz & Neumann, 1999) showed stronger inhibition by incongruent primes than facilitation by congruent primes. On the other hand, congruent and incongruent refer to prime-target relations, that is, primes as such are neither congruent nor incongruent. A pure prime-related effect should therefore be independent of congruence/incongruence or, put conversely: If the prime’s effect was different between congruent and incongruent trials, this would be evidence that prime-related and target-related activity are not independent from one another. Experiment 2 was intended to address this issue by adding a condition with neutral primes. By comparing the data from congruent and incongruent trials to this neutral condition, inhibition, and facilitation can be assessed independently.

To summarize, Experiment 1 has yielded two independent measures of the influence of masked primes on the motor system: The primes tend to elicit a motor response even if the target stimuli require the participant not to respond; and the LRP waveforms that can be attributed to primes seem to be independent of whether or not the target requires a response. The questions raised by the results of Experiment 1 are, first, why there was no incongruity dip, and second, whether the prime’s effect on the LRP was not only independent of whether the target was a go or a nogo target, but was also independent of whether or not a go target was congruent or incongruent. These two issues were addressed by Experiment 2.

## Experiment 2

One purpose of Experiment 2 was to determine whether the activation by the prime is equally large in incongruent and in congruent conditions. For that purpose, in addition to the congruent and incongruent conditions from Experiment 1, a third condition was introduced, the neutral condition. In this condition, the prime pair consisted of two distractor-like shapes, that is, it was not associated with either of the responses. By relating the results from congruent and incongruent priming conditions to this neutral condition, the relative strengths of the effects of congruent and incongruent primes could be determined.

Thus, data analysis involved two steps. First step was to calculate the influence of the prime from congruent and incongruent conditions. This procedure was identical to that of Experiment 1. Second, add/subtract this calculated prime-related activity to/from the activity measured in the neutral condition. This results in calculated congruent and calculated incongruent LRPs. The decisive step is the comparison of the calculated congruent and incongruent LRPs with the corresponding empirically measured LRPs. If they are identical, then the independence of the prime-related activity with respect to the direction of the target-related activity has been demonstrated.

To look into the origin of the absence of an incongruity dip in Experiment 1, we omitted the nogo condition. As discussed after Experiment 1, we reasoned that the dip should be absent if the activity caused by the prime starts simultaneously with that of the target, and that this might be the case if there is a nogo task, postponing response preparation until the target’s onset.

### Method

Thirty-five students (15 male, 20 female; mean age 24 years) at the University of Bielefeld were recruited for the experiment. All had normal or corrected-to-normal visual acuity. They participated in a single session of approximately three hours and were paid for their participation.

These were the same as in Experiment 1, with the following exceptions:

(1) There was no nogo condition.

(2) There was a neutral condition with two distractor-like shapes as primes.

(3) In addition, we used a condition with a target but without primes. (The data from this condition target alone will not be considered in the present analysis, since they were collected for a different purpose.)

Each participant served in one block of 800 trials, consisting of 200 congruent, 200 incongruent, 200 neutral, and 200 mask-alone conditions. Data were sampled at 250 Hz in epochs of 900 ms. In addition to the electrode sites of Experiment 1, data were also derived at the sites FP1, FP2, F3, F4, F7, F8, P3, P4, 01, 02, T3, T4, T5, and T6. Instead of Ag-AgCl electrodes, an electro cap from International Inc., Eaton, Ohio, was used.

### Results

#### Reaction times and error rates

None of the participants were excluded (see Results of Experiment 1 section). Out of all trials, 0.4% were excluded from analysis because RT either exceeded 1,000 ms or was faster than 100 ms. RTs in congruent, neutral, and incongruent conditions were 384 ms, 400 ms, and 424 ms, respectively. An ANOVA revealed a significant effect of conditions, *F*(2, 56) = 93,6, *p* < .001. Subsequent matched t-tests yielded a significant difference, *t*(28) = 11.0, *p* < .001 between congruent and neutral conditions as well as between neutral and incongruent conditions, *t*(28) = 9.7, *p* < .001. In 1% of the congruent and in 3.4% of the incongruent trials, participants made an error. In neutral conditions, this occurred in 1.1% of the trials. This congruent-versus-neutral difference was significant, *t*(28) = 5.1, *p* < .001, as was the neutral-versus-incongruent difference, *t*(28) = 4.4, *p* < .001.

#### Event-related potentials

**Figure 3.**
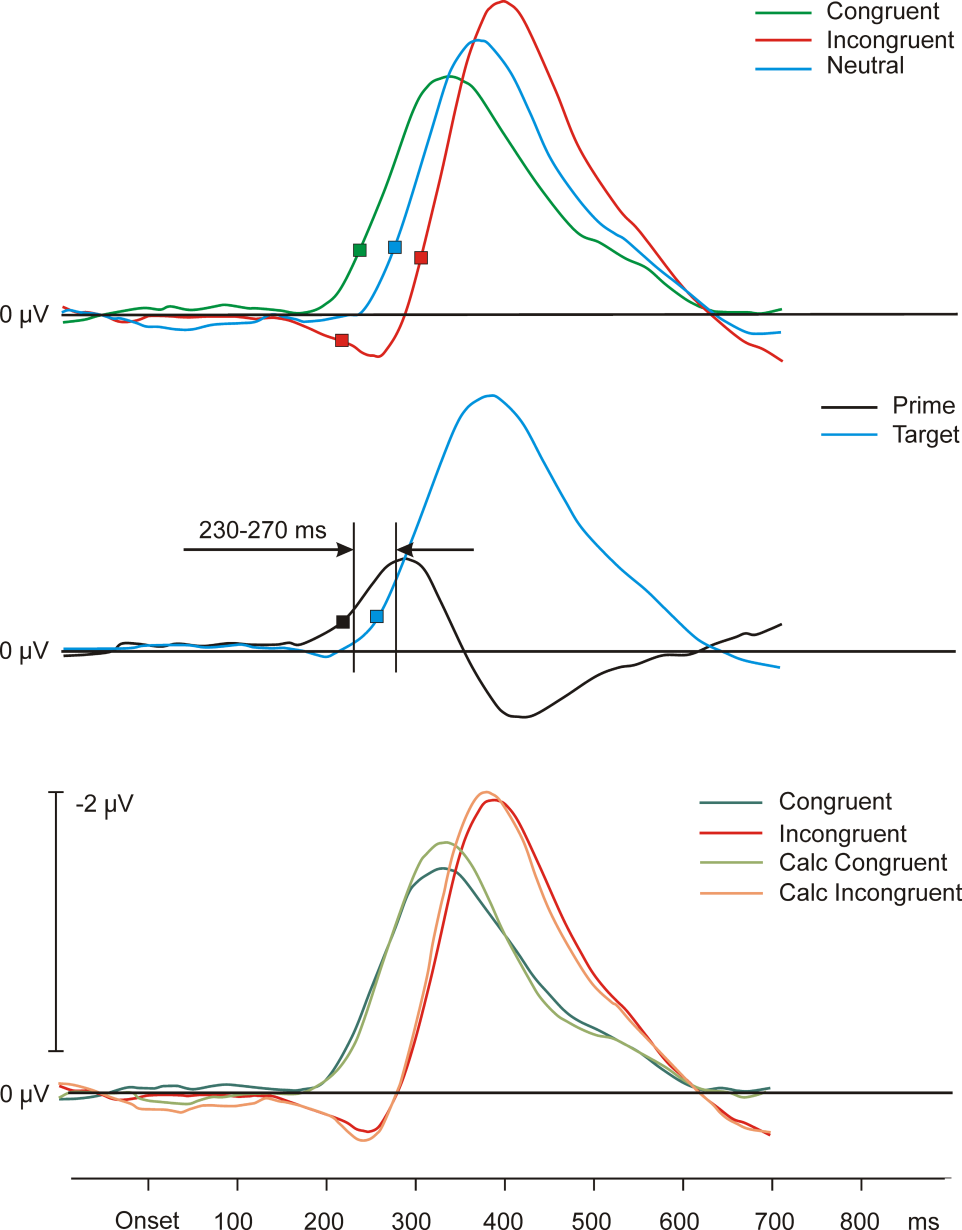
Upper panel: Grand average LRP waveforms of Experiment 2. Depicted are congruent, neutral, and incongruent conditions. Middle panel: Grand average waveforms of the calculated LRP effects across conditions of prime and target in Experiment 2. Lower panel: Directly measured congruent and incongruent conditions in comparison to the calculated congruent and incongruent conditions. The basis for the calculated conditions is the neutral condition. Calculated congruent and incongruent conditions result from the directly measured neutral prime activity plus/minus the prime activity averaged across congruent and incongruent conditions. Symbols stand for LRP onsets.

Out of all the trials, 28% were rejected as artifacts. Discarded trials were equally distributed across conditions. Figure 3 (upper panel) depicts the grand average LRP waveforms for each condition. The onsets of the congruent, neutral, and incongruent LRPs were at 230, 265, and 295 ms, respectively. Prior to the target-contralateral activation, a target-ipsilateral (or prime-contralateral) activation (dip) occurred in the incongruent condition (onset after 205 ms). Averaging across conditions to reveal the respective impacts of primes and targets resulted in the waveforms shown in Figure 3 (middle panel). The calculated prime LRP had its onset after 205 ms and the calculated target LRP after 245 ms. At the beginning of the lateralization, the calculated prime-related activity was stronger than the calculated target-related activity. Their mean activity was measured in a time window between 230–270 ms; prime -0.54 µV, target -0.21 µV, *t*(28) = 2.75, *p* < .01.

To determine whether the effects of congruent and incongruent primes were equal to one another, we performed the calculations described in the introduction for the present experiment: Prime-related activity was calculated as in Experiment 1. Additionally, over the whole course of the derivation, this calculated prime-activity in the congruent condition was added to the calculated prime activity measured in the neutral condition, and the calculated prime activity in the incongruent condition was subtracted from that in the neutral condition. These new estimates can be seen in Figure 3, in which they are compared to the empirically observed congruent and incongruent prime activities (i.e., those activities which were not added to or subtracted from the neutral prime activity).

### Discussion

Experiment 2 provides rather clear-cut answers to the two questions that had arisen from the results of Experiment 1. First, by introducing a neutral condition, we could independently estimate the relative strengths of the effects of congruent and incongruent primes in two different ways: with and without taking into account the prime activity from neutral trials. These two estimates were virtually identical to one another, strongly suggesting that the effect of a prime does not depend on whether or not it primes the same response as the target. Together with the finding from Experiment 1 that the prime’s effect is independent of whether a go or a nogo target is used, this rather unambiguously demonstrates that the activity caused by the prime is independent of the activity caused by the target.

In Experiment 1, we wondered why the usually robust incongruity dip was absent, and we reasoned that this might have been due to the presence of the nogo condition. This was fully confirmed by the results of Experiment 2. By omitting the nogo condition, we could reinstate the dip.

## Experiment 3

Experiment 1 showed that the prime-related activity calculated from the go conditions and the directly measured prime-related activity with the nogo target did not differ. Experiment 2 demonstrated that the directly measured lateralizations under congruent and incongruent conditions could be also created from the estimate that combined prime-related activity from the congruent and incongruent conditions with that from the neutral condition. In other words, the effect of a prime was the same whether it preceded a congruent or an incongruent target.

In Experiment 2, there was no nogo condition, because we suspected that due to the nogo condition, the response criterion was changed in such a way that the dip was absent in incongruent conditions. The independence hypothesis has to sustain, however, the absence of the dip. Even then, the congruent and incongruent course of the lateralization ought to be constructible on the basis of the course of the neutral condition plus/minus the lateralization by the activity of the prime. The nogo condition is re-introduced to reduce the dip. In Experiment 3, there are congruent, incongruent, neutral, and nogo conditions.

### Method

Thirty-two students (18 male, 14 female; mean age 25 years) at the University of Bielefeld took part in the experiment. All had normal visual acuity, they were all tested in one single session of approximately three hours, and they were paid for their participation.

These were the same as in Experiment 1, with the exception that also the neutral conditions as described in Experiment 2 were used. Besides the neutral condition from Experiment 2, there was a neutral condition consisting of two imperative, that is, target-shaped primes. Only this neutral condition will be considered in the following, because we considered a “neutral” condition consisting of two distractor-shaped primes as problematic in the present context, in which two visible distractors were used as a nogo stimulus. (Later conducted analyses, however, revealed that there were no differences between different neutral conditions in RTs and LRPs.) The number of trials was 120 per condition.

### Results

#### Reaction times and error rates

Nine participants were excluded from the analysis due to inadequate measurement or too many artifacts in the EEG recording (see Results of Experiment 1 section). Out of the trials, 0.3% were discarded as outliers. In congruent trials, RT was 393 ms, with 0.4% errors. In incongruent trials, RT amounts to 423 ms with 0.7% errors. In neutral trials, RT amounted to 411 ms with 0.5 % errors. The ANOVA for the RTs was significant, *F*(2, 44) = 26.5, *p* < .001. The ANOVA for the errors failed significance, *F*(2, 44) = 0.1, *p* > .80. Matched t-tests revealed significant differences for neutral versus congruent RTs; *t*(22) = 4.78, *p* < 0.001; and for incongruent versus neutral RTs, *t*(22) = 3.37, *p* < 0.01. False alarms in the nogo condition amounted to 0.5% of responses corresponding to the target-shaped prime’s location and 0.1% of non-corresponding responses. A matched *t*-test comparing between these conditions was significant, *t*(22) = 3.76, *p* = 0.001.

#### Event-related potentials

**Figure 4.**
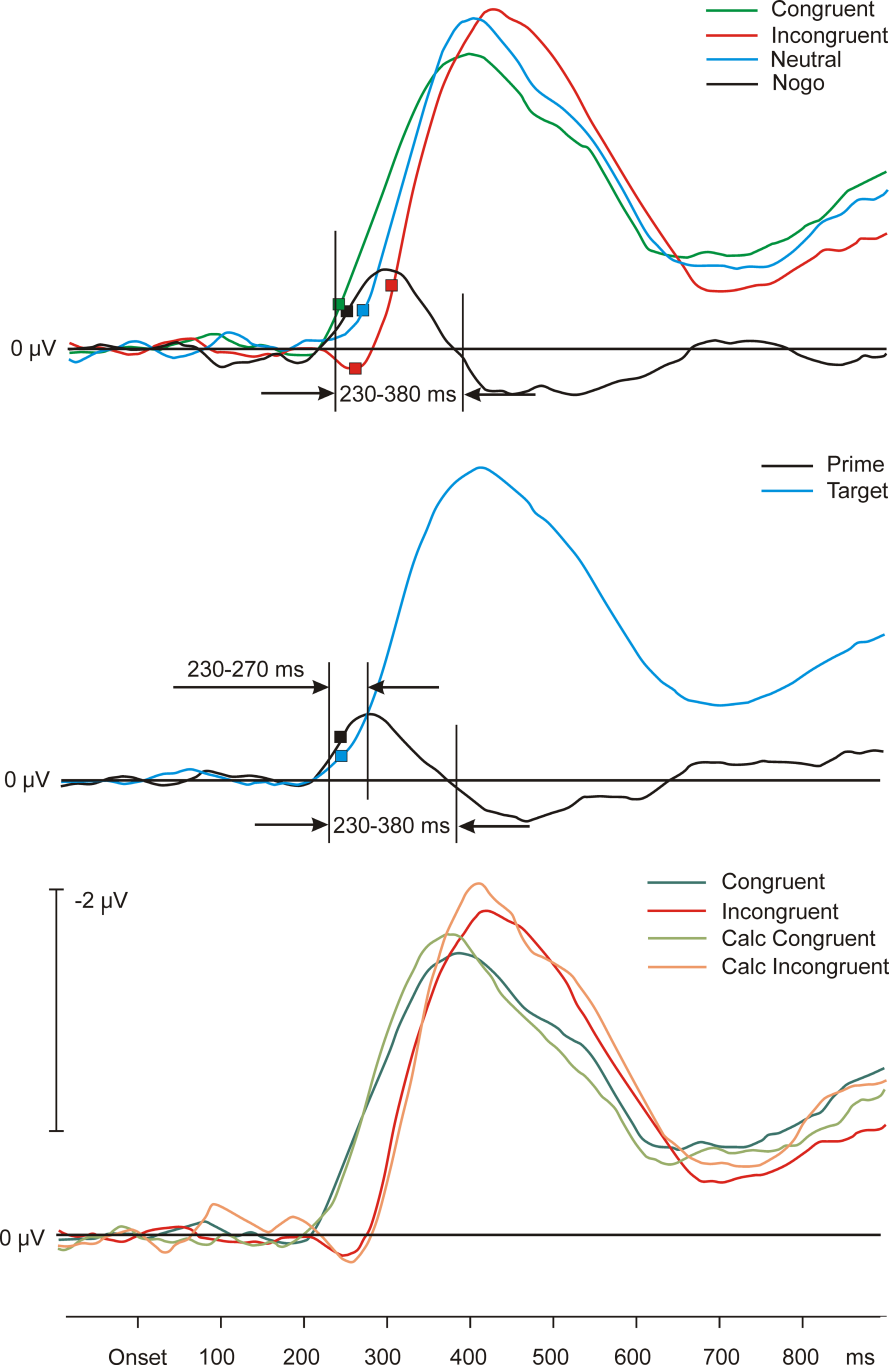
Upper panel: Grand average LRP waveforms of Experiment 3. Depicted are congruent, neutral, and incongruent conditions. Middle panel: Grand average waveforms of the calculated LRP effects across conditions of prime and target in Experiment 3. Lower panel: Directly measured congruent and incongruent conditions as compared to the calculated congruent and incongruent conditions. Basis for the calculated conditions is the neutral condition. Calculated congruent and incongruent conditions result from the directly measured neutral plus/minus the prime activity under the nogo condition. Symbols stand for LRP onsets.

From the data of the 23 participants, 15% of all trials were rejected as artifacts. Discarded trials were equally distributed across conditions. The averages are shown in Figure 4 (upper panel). The onsets of the LRP waveforms were 228, 260, and 292 ms for congruent, neutral, and incongruent conditions, respectively. The dip in the incongruent conditions was weak. Just three successive data points could be found in which the incongruent prime-induced LRP differs significantly from the zero activity baseline (onset 248 ms). The prime-related activity in the nogo condition started after 236 ms. Averages for different stimuli across conditions are depicted in Figure 4 (middle panel). The onsets of the target and the prime LRP were both at 236 ms. In the time window from 230–270 ms, mean activity of prime and target was compared and found not to significantly differ, prime -0.31 µV, target -0.17 µV; *t*(22) = 1.14, *p* > .15). The ascending slopes of the calculated prime-related and target-related LRPs are again virtually identical.

To find out whether the prime-related activities differed between go trials and nogo trials, we compared the average amplitudes of these conditions in a time window between 230–380 ms. These mean amplitude differences were found to be insignificant, go trials: -0.40 µV, nogo trials: -0.50 µV; *t*(22) = 0.84, *p* > .40.

Calculated congruent and incongruent conditions were determined as follows: Over the whole span of the potentials, the corresponding data of the prime under the nogo target were added to (incongruent condition) or subtracted from (congruent condition) the data of the neutral condition. Figure 4 (lower panel) shows the calculated activities from congruent and incongruent conditions as compared to the empirically observed activities in congruent and incongruent conditions. The match is striking, statistical analysis was dispensable.

### Discussion

Experiment 3 was a successful replication of Experiment 2’s main results and, thus, confirmed that prime-related activity is independent of target-related LRP activity. In particular, again there was no difference between the calculated congruent and incongruent LRP (in which we took into account the activity in the neutral baseline condition besides the prime-induced activity) and their empirically observed congruent and incongruent LRP counterparts. This finding of no difference corroborated the conclusion that the prime-evoked LRP under congruent and incongruent conditions was indeed independent of the target-evoked LRP. Otherwise, an interaction between the target-evoked LRP and the prime-evoked LRP in congruent and/or incongruent conditions should have shown up.

In the present experiment, weaker prime-evoked motor activation effects were to be expected; more precisely the absence of the dip in incongruent conditions was expected on the basis of its absence in Experiment 1, in which nogo target trials were also used. Contrary to this expectation, we found an incongruity dip in the current experiment’s incongruent condition, although the deviance was small and of a brief duration. In a nutshell then, the factor that determined the amount of time by which the onset of the prime-related LRP preceded that of the target-related LRP seems to be related to whether or not nogo target trials were included, but the factor was not identical to that manipulation.

Instead, the factor that seems to be responsible for the relative timing of prime-related LRPs is the utility of the prime for activating the finally required accurate motor response. This might be inferred from rank ordering of Experiments 1-3 with respect to the rate of trials in which the target countermanded the prime’s evoked motor activity: We found that LRP onset differences between prime and target followed the resultant prime utility. In Experiment 2, the prime’s utility was highest, and participants consequentially used the lowest criterion for response activation on the basis of the prime alone: Targets countermanded the prime’s motor activation in only the incongruent trials, that is, in only 25% of all trials. Therefore, the prime-related LRP had a substantial head-start relative to the target-related LRP, and an incongruity dip was observed. In Experiment 3, prime utility decreased, and the participants consequentially increased their criterion for activating a response on the basis of the prime alone: Using nogo targets in 1/5 of all trials but also neutral primes in 2/5 of all trials, the rate of trials in which targets countermanded the prime’s activated motor responses raised to 40% (1/5 incongruent trials + 1/5 nogo trials). Thereby, the head-start of the prime-related LRP relative to the target-related LRP was also reduced. Finally, in Experiment 1, prime utility was lowest, and consequentially the criterion for a motor activation on the basis of the prime alone was even further increased: Using nogo targets in 1/3 of all trials and incongruent prime-target sequences in another 1/3 of all trials, the rate of trials in which targets countermanded the prime’s evoked motor response increased to 66.7%. Under such conditions, no temporal precedence of prime-evoked LRPs over target-evoked LRPs whatsoever resulted, and consequentially, the incongruity dip was absent.

## Experiment 4

Consider once again the course of events in the different conditions of Experiment 1. A prime pair is presented that contains a target-like shape, and apparently this target-like prime activates a response that is seen in the LRP and, in some trials, results in an erroneous response. This activation seems to be the same whatever the response to the target. Even if no response to the target is given, prime-evoked LRPs can be observed. Also, whether prime-target sequences are congruent or incongruent does not matter for the prime-evoked LRP. Therefore, it might be expected that the prime’s motor activation effect also occurs if there is no target at all.

To test this prediction, in Experiment 4, the targets were omitted in an additional nogo condition. The primes thus became visible as a type of nogo stimuli. This status of being nogo stimuli, however, equally held true for masked primes, since, logically, observers could not know until 75 ms after the prime’s onset whether or not there would be a subsequent target. A comparison of the impact of masked and unmasked primes should therefore provide an especially conservative test of the independence of a prime’s motor activation effect from subsequent stimulus events. This test is conservative because of the impact of the prime’s utility for activating the finally required response that was suggested by a comparison of LRP onset differences between prime and target across Experiments 1-3. Under the present conditions, it is the case that the criterion for activating a response solely on the basis of the prime should be increased because this time, the prime itself sometimes countermanded its motor activation once it became visible.

### Method

Twenty-four students (13 female, 11 male; mean age25 years), all with accurate visual acuity, at the University of Bielefeld took part in the experiment. They were all tested in one single session of approximately 3 hr, and they were paid for their participation.

These were the same as in Experiment 1, with the exception that a further 160 nogo trials were added. These trials constituted an additional nogo condition. The experiment thus consisted of a congruent and an incongruent condition, plus a nogo condition with a prime pair containing one target–shaped prime followed by a mask pair of two distractors, and another nogo condition in which only the prime pair was displayed without a subsequent mask.

Participants were instructed to respond to the side at which the target appeared and to refrain from responding when the target was not shown. It was emphasized that the target was one of the larger stimuli (e.g., the large diamond) and that no response to the smaller prime in the (unmasked) cases was to be given.

### Results

#### Reaction times and error rates

The data of 6 subjects had to be excluded because of inadequate measurements or an excessive amount of artifacts (blinks and/or eye movements; see Results of Experiment 1 section). Out of the trials, 0.2% were discarded as outliers. In congruent trials, RT was 406ms, with 0.4% errors. In incongruent trials, RT amounted to 436ms, with 1.2% errors. Matched t-tests revealed significant differences for RTs, *t*(17)=7.2, *p*<.01, as well as for error rates, *t*(17)=3.0, *p*<.01. False alarms in the prime-only nogo condition amounted to 0.6% of responses to the side of the target-shaped prime and 0.1% of non-corresponding responses. For masked primes, these proportions were 2.4 and 0.3%, respectively. A two-way repeated factors ANOVA of the arc-sine transformed false alarm rates revealed significant main effects of masking (masked vs. unmasked primes), *F*(1, 17)=18.0, *p*<.01, and side of the target-shaped prime (equal vs. unequal to the response side), *F*(1, 17)=14.8, *p*<.01, as well as a significant interaction, *F*(1, 17)=8.7, *p*<.01.

#### Event-related potentials

**Figure 5.**
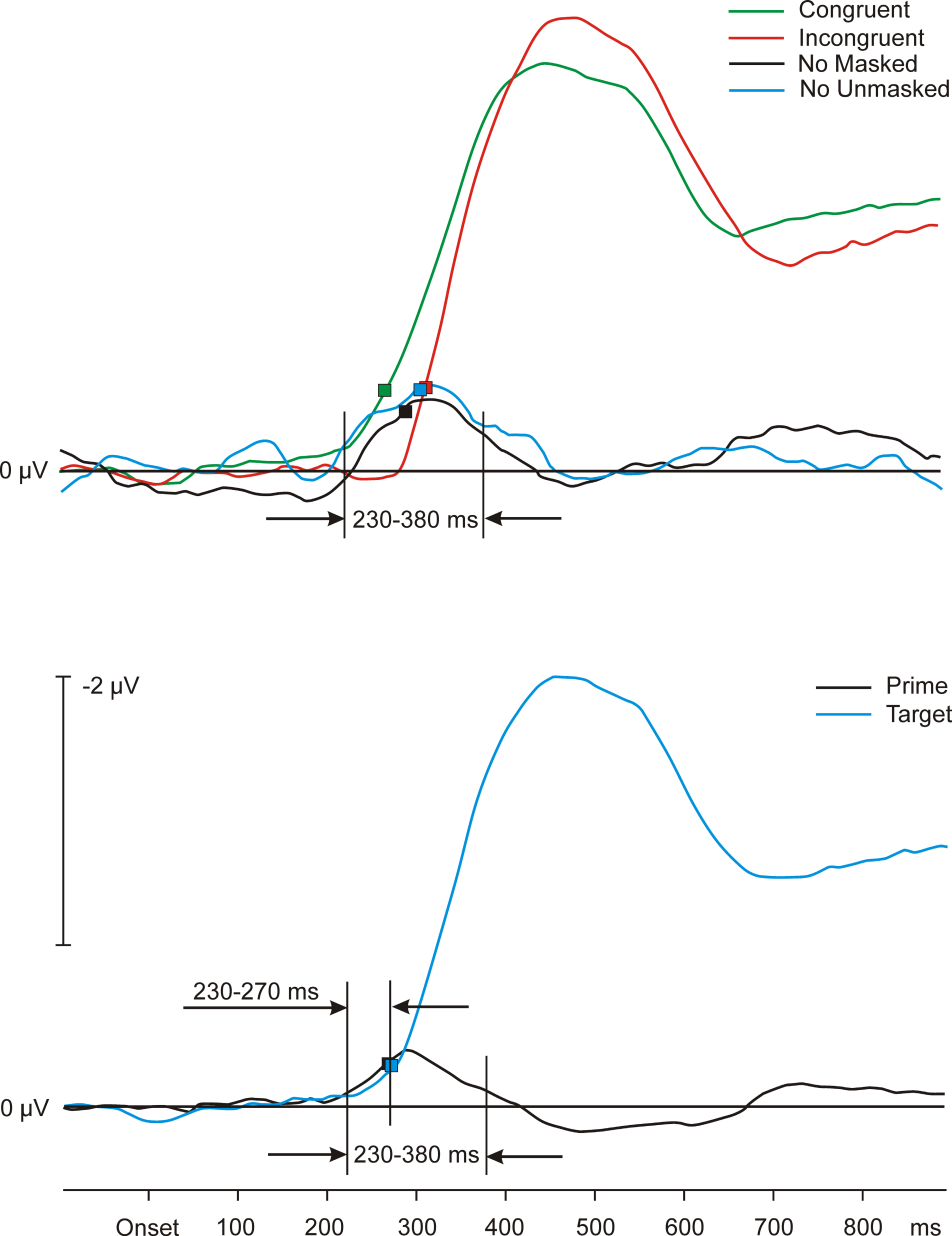
Upper panel: Grand average LRP waveforms of Experiment 4. Depicted are the two nogo conditions consisting of masked and unmasked prime. Furthermore, the congruent and the incongruent conditions are shown. Lower panel: Grand average waveforms of the calculated LRP effects across conditions of prime and target in Experiment 4. Symbols stand for LRP onsets.

From the data of the remaining 18 participants, 19% of all trials were rejected as artifacts. Discarded trials were equally distributed across conditions. LRP data that were suitable for analysis are shown in Figure 5 (upper panel). The onsets of the LRP waveforms were 260, 308, 304, and 284ms for congruent, incongruent, prime-only nogo, and masked-prime nogo conditions, respectively. The incongruity dip was absent. Averages for each stimulus across conditions are depicted in Figure 5 (lower panel). The onset of the target-induced LRP was at 268ms and the onset of the prime-induced waveform at 260ms. To determine whether onset activity between prime LRPs and target LRPs differed, we compared the ascending slope of the mean lateralized activities in a time window between 230–270 ms. There were no significant differences in that time window, prime: -0.18 V, target: -0.11 V; *t*(17) = 0.80, *p* > .40. To test for differences between the prime-evoked activity in masked prime versus visible prime (nogo prime) conditions, we compared the corresponding mean values in a time window of 230–380 ms. Again, we found no significant differences; masked congruent and incongruent prime: -0.27, masked prime preceding a nogo target: -0.40, visible prime (nogo prime): -0.51; *F*(2,34) = 1.0, *p* > .35.

### Discussion

The masking of the prime is not decisive for the LRP effect of the prime. Both nogo conditions revealed essentially the same activation in their respective LRPs. How can this be understood? The prime has two relevant properties: shape and size. Participants can decide about the side of the response (responding hand) on the basis of visual shape. However, only on the basis of size can the participants decide whether to respond or not to respond. Thus, it suffices that shape can be discriminated before size for a priming effect. Once shape is discerned before size, for instance because shape features differ to a larger degree than size features, the prime shape happens to activate a response reflected in the LRP that can only later be inhibited by stimulus size.

This sequence of events, with shape discrimination preceding size discrimination, however, fails to account for the false alarms in the two nogo conditions. These showed a remarkable pattern: Participants responded more often in the masked condition than in the prime-only condition. In both conditions, the false alarms corresponded to the side of the target-shaped prime in a majority of trials. However, the difference between corresponding and non-corresponding false alarms was significantly higher in the masked condition.

What makes the picture even more perplexing is the fact that the differences in the rates of corresponding against non-corresponding false alarms of masked versus visible primes were not also reflected in associated LRP differences between the conditions. Apparently, the number of false alarms cannot be predicted on the basis of the prime’s LRP alone. If, as the LRP strongly suggests, masking does not influence the prime’s response activation, the false alarm differences must be explained by assuming another origin of the effect. We think that different response thresholds in masked and unmasked conditions might readily account for the differences. Shifting the threshold of minimal LRP activity necessary for triggering a response toward lower values in masked than in visible conditions could be a way to reconcile similar LRP effects with different false alarm rates. This fits nicely with an explanation of the masked priming effect by an accumulator model (Vorberg, Mattler, Heinecke, Schmidt, & Schwarzbach, 2004). The model assumes accumulation processes for each of two possible responses, fed by sensory input from primes (and targets or masks). When the difference between the two accumulated response activations reaches a predefined threshold or criterion, one of the responses is triggered. In the present case, it seems as if this threshold was either raised in the visible prime condition or lowered in the masked prime condition.

## General Discussion

In four experiments, we studied the response activation by the prime in the metacontrast paradigm (Klotz & Neumann, 1999; Klotz & Wolff, 1995; Neumann & Klotz, 1994). In particular, we examined the independence of the response activity caused by the prime of the response activity caused by the target. To that end, the response-related lateralized activity was measured over the motor cortex.

In Experiment 1, congruent and incongruent conditions were presented. Moreover, in a target-nogo condition, the prime could be followed by a mask consisting of two distractors to which the participants could not respond. From the congruent and incongruent conditions, we then derived the prime-related activity under go conditions: Across congruent and incongruent conditions, an LRP was derived reflecting contra- versus ipsilateral activity differences (lateralized potentials) with respect to the prime side. This activity was called the prime-related activity because target-related lateralized activity was cancelled out. Importantly, it turned out that prime-related lateralized activities were equal under target-go and target-nogo conditions. This suggests an independence of the motor activation by the prime regardless of whether a go or a nogo signal was used in the target-distractor display. In other words, the prime-induced response activation is not influenced by the target-induced response activation. This finding is in line with an assumed sequence of response-activation effects of the response activation by the prime temporally preceding that of the target.

The procedure in Experiment 1 does not allow us, however, to compare sizes of the prime-related lateralized activity between congruent and incongruent conditions. Thus, Experiment 1 left open the question whether the spatial prime-target relation (i.e., whether it was congruent or not) impacted on the prime-induced response activation. This was studied in Experiments 2 and 3. If the lateralized activity evoked by the prime is independent of the lateralized activity evoked by the target, then the prime-related LRP should be equally large in congruent and in incongruent conditions. To test this prediction, the following procedure was used: Besides the congruent and incongruent conditions, a neutral condition was introduced. In the neutral condition, the prime pair consisted of two distractor-shaped stimuli. In these experiments, we next added the prime’s evoked activity (derived as above) to the activity measured in the neutral condition and subtracted the prime’s activity from that in the neutral condition. We then compared the resulting LRPs to the LRP in congruent and incongruent conditions. These LRPs were the same, regardless of whether they were computed relative to the neutral baseline condition or whether they were collected empirically. Consequently, the prime-related activity is independent of whether the target-distractor required a response or not, and it is also independent of whether the response to the target is the one that has already been activated by the prime or whether it is the alternative response as compared to the one activated by the prime. One might expect that at least the interruption by the mask (regardless of whether this is the go target or the nogo mask) has an impact on the response activation by the prime. Even this is not the case, however, as was shown in Experiment 4. The prime exerted its LRP effect, and this was not influenced by the trailing masks or targets.

 These findings corroborate the assumption put forward by Schmidt and colleagues (Schmidt, Niehaus, & Nagel, 2006; Vath & Schmidt, 2007) that prime-evoked activity precedes target-evoked activity and, thus, escapes the latter at least during the first 100 ms of stimulus processing or so, that is, during the so-called feed-forward sweep of visual stimulus processing (cf. Lamme & Roelfsema, 2000). Schmidt et al. (2006) refer to this notion as a “rapid chase” between the prime-evoked response activation and that evoked by the subsequent target. 

A comparison of the relative timing of the onsets of prime-evoked and target-evoked LRPs across Experiments 1 to 3 of the present study led to another significant observation. The time by which the prime-evoked LRP preceded the target-evoked LRP was evidently dependent on the prime’s utility for activating the finally required response. Head-starts of prime-evoked LRPs over target-evoked LRPs were most pronounced where the prime’s utility was highest, and prime-evoked responses only relatively rarely had to be countermanded by the target-evoked responses (Experiment 2). Decreasing the utility of the prime for activating the finally required response also decreased the temporal precedence of onsets of prime-evoked LRPs over target-evoked LRPs (Experiments 1 and 3).

In conclusion, the participants evidently had some control over the time at which the response activation by the prime was fetched. That means that participants set up top-down controlled settings for processing of stimulus features in advance of the stimuli and in accordance with the utility of the primes. As a consequence of this strategic criterion setting, the onset of the sampling of response-related visual evidence was shifted toward or away from the onset of the visual stimulus. These findings neatly complement other recent evidence for the possibility that participants exert top-down control over the processing of subliminal visual input (cf. Ansorge, 2004; Ansorge & Heumann, 2006; Ansorge & Neumann, 2001, 2005; Eckstein & Perrig, in press ; Kunde et al., 2003; Schlaghecken & Eimer, 2004). The finding also makes clear why it is crucial for the masked priming effect that the onset of a masked prime can be successfully anticipated in time (cf. Kiefer & Brendel, 2006; Naccache, Blandin, & Dehaene, 2001).

 Another finding of interest concerned the prime-related lateralized activity: This activity was the same whether primes were masked or visible. This is in line with several findings. Vorberg et al. (2004), for example, varied the prime-target SOA in a very similar paradigm. They found a linear increase of the congruence-incongruence effect in a choice-reaction task but a quite different time course of prime visibility: The discrimination performance remained at a chance level. In other conditions, discrimination performance even followed the well-known inverse u-shaped function relating prime-mask interval to prime visibility, and still the RT congruence-incongruence effect linearly increased with the prime-target interval (cf. Schmidt & Vorberg, 2006). Thus, the strength of response priming by masked primes and their visibility were dissociated in these studies. Recent findings from our own laboratory lend further support to the notion that, under appropriate conditions, masking or visibility does not modulate a prime’s effect, in that case its potential to capture attention as evident in temporal order judgments (Scharlau & Neumann, 2003). 

Finally, of note is the LRP activity evoked by the primes under masked and visible priming conditions was similar, but the rates of false alarms to the side of the target-shaped prime were much higher in masked than visible priming conditions. We consider this pattern of false alarm rates to be evidence for a shifting of the criterion or threshold for giving one of the responses, with this threshold being decreased under masked relative to visible priming conditions. Also, the fact that the LRP amplitude was unaffected by visibility is well in line with the assumption of the accumulator model of the masked priming effect (Vorberg et al., 2004), according to which a second model parameter different from the threshold or criterion parameter accounts for response activation: This second criterion-independent parameter is the drift rate by which response activation for each of two alternative responses accumulates over time. In other words, it is our contention that the drift rate is reflected in the LRP amplitude and that the threshold or criterion is reflected in the false alarm rates. In the area of masked priming, the independence of drift rate and criterion from one another is a new finding. However, given that this sort of independence between drift rate and threshold has been found in a variety of two choice-reaction tasks (cf. Ratcliff, Van Zandt, & McKoon, 1999), this new finding is not unprecedented. To conclude, however, future research should aim to confirm this basic observation and to explore it in more detail.
